# Development of the Tonsil Microbiome in Pigs and Effects of Stress on the Microbiome

**DOI:** 10.3389/fvets.2018.00220

**Published:** 2018-09-19

**Authors:** Luis Carlos Pena Cortes, Rhiannon M. LeVeque, Julie A. Funk, Terence L. Marsh, Martha H. Mulks

**Affiliations:** ^1^Comparative Medicine and Integrative Biology Program, College of Veterinary Medicine, Michigan State University, East Lansing, MI, United States; ^2^Department of Microbiology and Molecular Genetics, Michigan State University, East Lansing, MI, United States; ^3^Facultad de Ciencias Agrarias, Universidad de Pamplona, Pamplona, Colombia; ^4^Department of Large Animal Clinical Sciences, Michigan State University, East Lansing, MI, United States

**Keywords:** microbiome, tonsil, pig, development, stress

## Abstract

Tonsils, lympho-epithelial tissues located at the junction of the oropharynx and nasopharynx, play a key role in surveillance, colonization, and persistence of inhaled and ingested pathogens. In pigs, the tonsils are a reservoir for numerous bacteria and viruses, including host-specific pathogens and potential zoonotic pathogens as well as commensal organisms. However, there are no in depth studies of the development of the tonsillar microbiome in pigs, or any mammal, over time. The goal of this study was to follow the development of the tonsil microbiome in healthy pigs from birth to market weight. Samples were collected using tonsil brushes from 16 piglets (4 each from 4 sows) at newborn, 1, 2, 3, and 4 weeks of age, and from 8 of those piglets at 6, 8, 10, 12, 16, and 19 weeks of age. Bacterial DNA was isolated from each sample and 16S rDNA genes were amplified and sequenced. Sequence analysis showed that members of the *Streptococcaceae, Pasteurellaceae*, and *Moraxellaceae* were present at all time points and represent the three most abundant families identified. Other community members appeared transiently or increased or decreased significantly with disruption events or stress. We observed four significant shifts in the tonsil community that coincided with well-defined disruption events: weaning plus addition of Carbadox plus movement to the nursery at week 3, removal of Carbadox and addition of Tylan at week 5, removal of Tylan and habitat change at week 9, and habitat change at week 16. Weaning triggered a bloom of *Streptococcaeae* and decrease of *Moraxellaceae*. The shift from Carbadox to Tylan led to reduction in *Proteobacteri*a and *Streptococcaceae* but an increase in other *Firmicutes*, accompanied by a dramatic increase in community richness. Cessation of Tylan coincided with a return to a less rich community, and a bloom in *Clostridiales*. The final shift in habitat was accompanied by a decrease in *Clostridiales* and increase in *Proteobacteria*. The tonsillar microbiome of older pigs resembled the previously described mature core tonsillar microbiome. This study demonstrates a temporal succession in the development of the pig tonsillar microbiome, and significant community shifts that correlate with disruption events.

## Introduction

Numerous bacteria and viruses can access the host using the oropharynx and nasopharynx as portals of entrance. One of the first lympho-epithelial tissues these organisms encounter is the tonsils, located at the junction of these two major portals of entry. Tonsils play a key role in immunologic surveillance of pathogens that access the host via these routes and in the initial process of pathogen-host colonization (1). Tonsils frequently serve as a reservoir of host-specific pathogens as well as zoonotic pathogens highly transmissible to humans, such as *Streptococcus suis, Salmonella enterica*, and swine influenza virus (2). Multiple pathogenic microorganisms, including bacteria and viruses, are regularly isolated from tonsils of asymptomatic animals. Pathogens residing in the tonsils can spread systemically or be transmitted to other animals including humans, with such transmission often triggered by stressful conditions such as transport (3). The resident tonsillar microbiome likely interacts with incoming pathogens, inhibiting colonization via competitive exclusion (4–7) as well as functioning to regulate immune homeostasis that is critical in providing resistance to infection (8, 9).

There are only limited numbers of studies addressing the tonsillar microbiome in humans or pig (10–17), in contrast to the growing number of studies on the intestinal microbiome in different species. Studies have suggested a gradual and sequential process in the development of the intestinal microbiome in humans and animals (18–21), where certain taxa persisted and became stable while others were acquired over time or only transiently. Further, microbiome development has been suggested to be based on specific bacterial interactions and not on random assembly of microorganisms (18). Intestinal microbial communities tended to achieve an adult-like profile as time progressed (18, 20). This trend was seen despite the fact that during development there were significant shifts in the structure of the population (20) as well as in the diversity (21), and many of these shifts were associated with life events or stresses such as diet changes and antibiotic treatment (18).

It has been demonstrated that common management practices such as the use of antibiotic treatments can significantly affect microbial communities and predispose the host to infections (22). However, the microbiota also can be shifted toward a microbial community that would protect the host from potential infections, as in the case of altering the intestinal microbiota through fecal microbiota transplantation (6). Despite the relevant role that the microbiota can play in maintaining good health status in the host and the key role played by the tonsils in both the respiratory and gastrointestinal tracts, there is a lack of knowledge about the development of tonsillar microbial communities of pigs or of the effects of stresses such as diet changes or antibiotic usage on the development, composition, and diversity of these communities.

Two studies have described the normal tonsillar microbiome in finishing (13, 14) and a third has described the metabolically active microbiome of slaughter pigs (15). The core tonsil microbiome in 18–20 week old grower-finisher pigs was comprised of members of the families *Pasteurellaceae, Moraxellaceae, Streptococcaceae, Fusobacteriaceae, Veillonellaceae, Enterobacteriaceae, Neisseriaceae*, and *Peptostreptococcaceae*, as well as the order *Clostridiales* (14). Whether a successional process is involved in how this core microbial community in the adult tonsils is established and develops over time and what role it plays in the acquisition and carriage of pathogens and thus in host health and disease is not known at this time.

We recently completed a study of the composition and development of the tonsil microbiome in piglets from birth up to 4 weeks of age (16). The tonsil microbial communities initially clustered by litter, but then converged by 3 weeks of age, regardless of litter or housing. These communities were comprised mainly of microorganisms acquired from the sow vaginal tract and teat skin, with a sequential succession observed over time. The combined stress of weaning, shift in food and housing, and addition of the growth promoter Carbadox® at 3 weeks of age led to a major shift in the microbiome at 4 weeks of age.

The goal of the current study was to extend this study, utilizing high-throughput sequencing of 16S rRNA genes to follow and describe the development of the tonsillar microbial communities in pigs through market age. This characterization of the development of the swine tonsillar microbiome lays a base for future studies that judiciously manipulates this microbiome to reduce pathogen load and improve overall animal health.

## Materials and methods

### Animals

This study and all animal procedures were approved by the Michigan State University Institutional Animal Care and Use Committee. The pigs used in this study were from a high health status farrow-to-finish herd with ~200 sows, housed at the Michigan State University Swine Teaching and Research Center. Relevant medical history for this herd included no recent respiratory disease; freedom from *Actinobacillus pleuropneumoniae, Mycoplasma hyopneumoniae*, and porcine respiratory and reproductive syndrome virus (PRRSV); a recent outbreak of porcine epidemic diarrhea virus (PEDV), under control prior to this study; and routine vaccination against erysipelas (*Erysipelas rhusiopathiae* bacterin ER Bac Plus, Zoetis Inc, Kalamazoo MI, administered intramuscularly) and porcine circovirus type 2 (Porcine Circovirus Vaccine, type 2, killed baculovirus vector, Ingelvac Circoflex, Boehringer Ingelheim Vetmedica, Inc, St. Joseph MO, administered intramuscularly). Four crossbred sows (Yorkshire X Hampshire) of different parity (number of pregnancies) were selected for this study and included sow 1700 (first parity), sow 1631 (second parity), sow 1445 (fifth parity) and sow 1711 (tenth parity). Four randomly selected piglets from each of the four sows were sampled within a period no longer than 8 h after birth (newborn) and the same piglets were sampled subsequently at 1, 2, 3, 4, 6, 8, 10, 12, 16, and 19 weeks of age.

**Figure 1:**
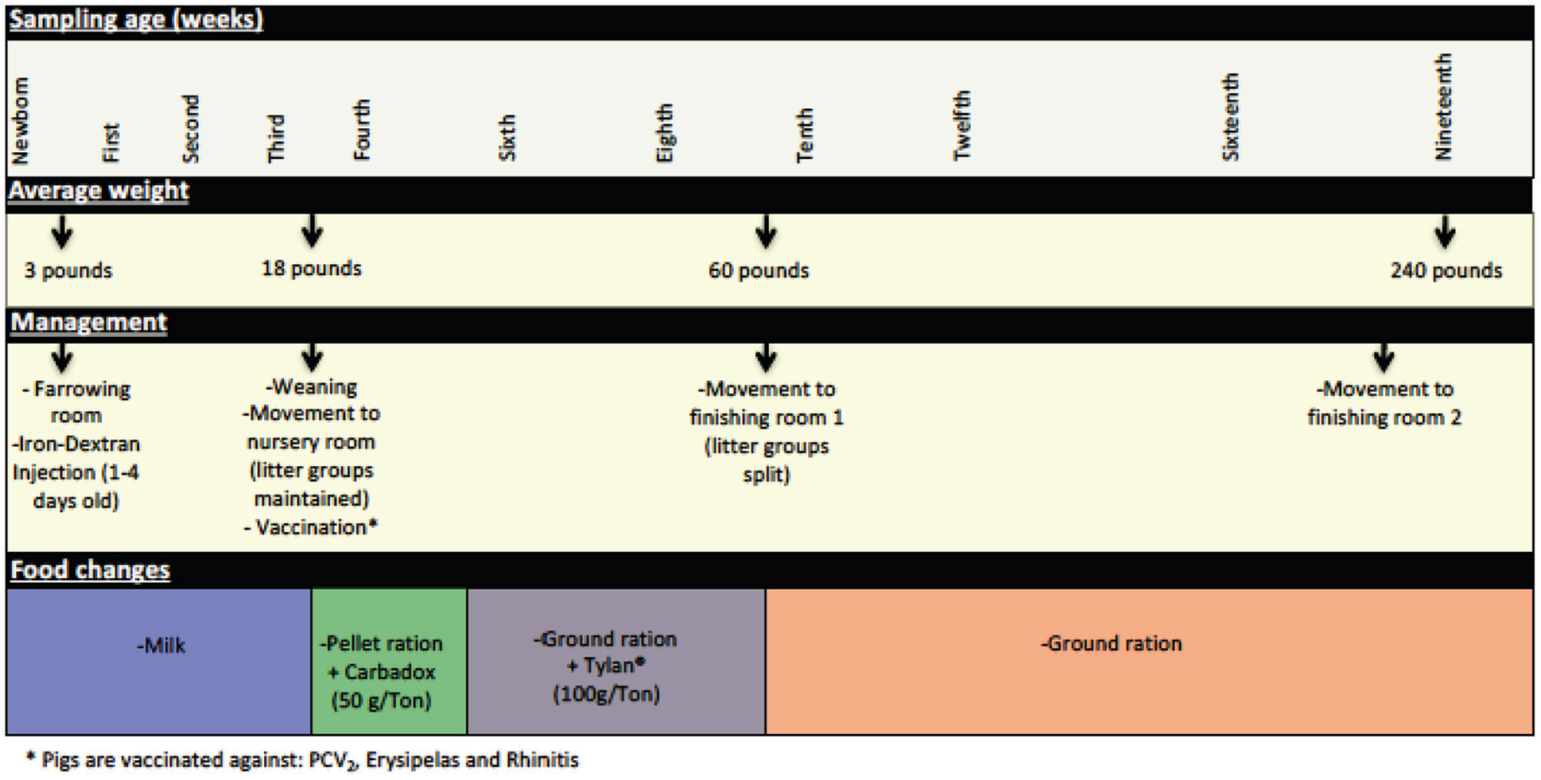
Significant management practices at the swine farm during the life of the pigs in this study. General management features experienced by the pigs during their life at the farm are depicted here, including changes in feed, use of in feed antibiotics, and movement to new housing.

Newborn piglets received a single intramuscular injection of Iron-Dextran (100 mg ANEM-X 100, Aspen Veterinary Resources, Ltd, Liberty MO) during their first week of life. Between the third and fourth weeks of age (21–24 days—average weight 18 pounds) piglets were weaned, vaccinated, and moved from the farrowing room where they were housed with the sow and littermates to a nursery room, with litters maintained as pen mates. At this time the piglets were weaned from milk to a solid pellet ration diet (Pig 1300®, Akey Nutrition, Brookville OH) supplemented with Carbadox® at a dose of 50 g/ton. Two weeks after being moved to the nursery facility (at 5 weeks of age), Carbadox® supplementation was removed from the feed, and food was changed to a ground ration supplemented with Tylan® at a dose of 100 g/ton. At ~9 weeks of age (63–67 days—average weight 60 pounds), piglets were moved to a finishing room and were assigned to different pens based on criteria such as gender and weight; separation by litter was no longer maintained. At this time, Tylan® supplementation was discontinued and a ground ration without supplementation was provided. Finally, at ~eighteen weeks of age, piglets were moved again to another finishing room (with another mixing of prior penmates) where they remained until being moved to the slaughterhouse (average weight 240 pounds). These management practices are summarized in Figure 1.

A total of 128 pig tonsil microbiome samples from pigs at birth through market age were sequenced and analyzed. Of these, 64 samples were collected from piglets before weaning (newborn—third week), which included samples from 16 piglets (4 per sow) at each time point. In addition, 64 samples were collected from pigs after weaning, which included samples from 16 piglets at week 4 and 8 piglets (2 per sow) at all subsequent time points (Table 1). In a previous study (16), we found a strong litter effect on the tonsil microbiome that disappeared by 3 weeks of age; therefore, we analyzed 4 pigs per litter initially but reduced this number to 2 pigs per litter (8 in total) for subsequent sampling times.

**Table 1:** Samples processed by sampling time and litter.

	**Sow ID**	**1700**	**1631**	**1445**	**1711**
	No. sow parity	1	2	5	10
	Litter members	10,11,12,13	15,16,17,18	36,39,40,42	22,23,24,26
ID	Sampling time	Number of samples analyzed per sampling time			
A	Newborns	4	4	4	4
B	First week	4	4	4	4
C	Second week	4	4	4	4
D	Third week	4	4	4	4
Total samples before weaning		16	16	16	16
E	Fourth week	4	4	4	4
F	Sixth week	2	2	2	2
G	Eighth week	2	2	2	2
H	Tenth week	2	2	2	2
I	Twelfth week	2	2	2	2
J	Sixteenth week	2	2	2	2
K	Nineteenth week	2	2	2	2
Total samples after weaning		16	16	16	16

### Collection of microbiome samples

Tonsil brushes developed by our group and validated in previous studies (14) were used to collect tonsil microbiome samples from sows and larger piglets, while Cytosoft™ cytology brushes (Medical Packaging Corporation, Camarillo, CA) were used for smaller piglets (16). Collection and storage of samples was as previously described (14).

#### Isolation of community DNA

Extraction of community DNA from samples was performed using a PowerSoil DNA Isolation Kit and PowerBead tubes (MoBio Laboratories, Carlsbad, CA) as previously described (13, 14, 16).

#### Illumina sequencing and sequence analysis

Sequencing was performed at the MSU Research Technology Support Facility (RTSF) as previously described (16). Negative controls consisting of DNA-free water or MoBio C6 reagent were used as “blank library controls” (16) and included in each sequencing run. Briefly, uniquely indexed primers were used to amplify the V4 region of the 16S rRNA gene from the community DNA, as described by Caporaso (23). A SequalPrep normalization plate (Invitrogen) was used to normalize the amplification products, which were then pooled and the reaction cleaned using AMPure XP beads. The pooled sample was sequenced on an Illumina MiSeq v2 flow cell using a 500 cycle v2 reagent kit (PE250 reads). Base calling was performed using Illumina Real Time Analysis Software (RTA) v1.18.54 and output of RTA demultiplexed and converted to FastQ files using Illumina Bcl2fastq v1.8.4.

The open-source, platform-independent, community-supported software program mothur v.1.35.0 (http://www.mothur.org) (24) was used for amplicon analysis. Raw sequencing data was processed according to the mothur standard operating procedure (http://www.mothur.org/wiki/MiSeq_SOP) (25) and aligned using the mothur-formatted version 123 of Silva 16S ribosomal gene database (26). After sequences were classified, all sequences classified as Chloroplast, Mitochondria, unknown, Archaea, or Eukaryota were removed from the data set. Subsampling at 7000 sequences per sample was done, followed by a preclustering of the sequences and removal of chimeric sequences using a mothur formatted version of the Ribosomal Database Project (RDP) training set version 14 and uchime, based on mothur protocol. A cutoff of ≥97% sequence identity was used to classify sequences into Operational Taxonomic Units (OTUs). Singleton and doubleton reads were removed before the final analysis. For the final analysis of the data, samples were subsampled to 5179 reads per sample. The full data set analyzed and the mother code used are available as a supplemental file at https://figshare.com/s/7147a352573045d7cf5c.

The samples for piglets from birth through 4 weeks of age were a subset of a larger set of samples used in a prior study (16).

### Diversity and statistical analysis

A clustering cutoff of 3% for the processed sequences was used in the statistical analysis. Mothur output files were used to estimate alpha diversity (sobs) and beta diversity indexes, as well as representative sequences, all of which were calculated in mothur v.1.35.0 (http://www.mothur.org) (24). PAST3 (Version 3.14; http://folk.uio.no/ohammer/past/) was used for statistical analysis of the samples. FigTree (Version 1.4.3; http://tree.bio.ed.ac.uk/software/figtree/) was used for construction of dendrogram figures. The area of the ellipses for the two dimensional scatter plot was measured using ImageJ (27). RStudio (Version 0.99.446; https://www.rstudio.com/) and libraries: gplots (https://CRAN.R-project.org/package=gplots) were used to generate heatmaps. Inkscape 0.91 (https://inkscape.org/en/download/mac-os/), was used to process images and edit labels. Taxonomy tables and OTU plots were generated in Microsoft® Excel® 2011, where the analysis of samples was done with data that represented higher than 0.1% of the total reads for the samples analyzed.

### Availability of supporting data

Raw sequence data and metadata is available at NCBI database (SRA accession number: SRP144702). Reviewer / collaborator link to metadata, valid through 8-8-18: ftp://ftp-trace.ncbi.nlm.nih.gov/sra/review/SRP144702_20180508_081000_cb5ae17636e975f9bf71ddf5bc542075.

## Results

### Management practices are related with changes in population diversity

A total of 128 tonsil samples for microbiome analysis were collected at 11 time points during the life of the pigs in this study. Some of the sampling times were chosen specifically to represent times associated with management practices significant in the life of the pigs, including immediately prior to and after weaning, alteration in feed and in-feed growth promoters, and movement to new rooms (Figure 1).

Analysis of the alpha diversity (species richness at a specific site) of the tonsil microbiome, as measured by the total number of species observed (sobs), and the relation with the different changes experienced by the pigs during their life showed that the alpha diversity varied widely (Table 2). For newborn piglets, the average value of sobs was 110. The average sobs value decreased steadily in the following weeks (first to third week), dropping to a value of 83. This was accompanied by a marked decrease in the standard deviation to 24, indicating that the microbiome became very similar in all pigs by 3 weeks of age. In contrast, from week 4 to 10 there was a substantial increase in diversity that coincided with specific challenging events experienced by the piglets. Between the third and fourth week, the piglets were weaned, and at the same time they were moved to a nursery room, vaccinated and their diet was changed. These changes were reflected in a slight increase in the average and maximum sobs as well as the standard deviation. However, the biggest change in diversity occurred during the period where Carbadox® was removed from the diet and Tylan® was supplemented. Diversity increased to over three times the previous registered values for average sobs. Conversely, the removal of Tylan®, accompanied by the transfer of pigs to a finishing room where they were no longer segregated by litter, led to a trend of decreasing diversity. By week 19, this progressive decrease in the diversity led to a value of average sobs of 108. Overall, there was a pattern demonstrating that extended time under constant conditions led to fewer sobs and reduced standard deviation.

**Table 2:** Number of observed OTUs^a^ (sobs^b^) during the different sampling times.

**Sampling time**	**No. Samples analyzed**	**Average sobs**	**Min sobs^c^**	**Max sobs^d^**	**Standard deviation sobs**
Newborn	16	110	26	376	100.6
First week	16	104	37	223	58.1
Second week	16	107	49	242	59.0
Third week	16	83	57	132	23.8
Weaned, Moved to nursery, Vaccinated, Carbadox supplementation.					
Fourth week	16	111	53	175	38.7
Carbodox removed, Tylan supplementation added					
Sixth week	8	368	215	434	69.8
Eighth week	8	355	276	408	46.5
Tylan removed, Moved to finishing room 1, litters split					
Tenth week	8	257	123	377	96.6
Twelfth week	8	139	53	234	64.1
Sixteenth week	8	132	83	177	35.7
Moved to finishing room 2					
Nineteenth week	8	108	73	178	35.2

a *OTU, Operational Taxonomic Unit*.

b *sobs, total number of species observed, measured as the number of observed OTUs*.

c *Min sobs, Minimum number of observed sobs*.

d *Max sobs, Maximum number of observed sobs*.

### Challenging management conditions during development of the pigs generated disruption in the microbiome

**Figure 2:**
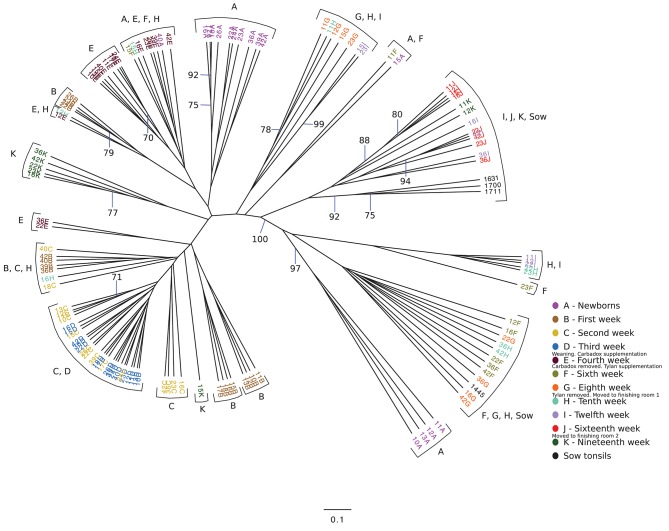
Unrooted Bray-Curtis dendrogram for all sampled weeks. The dendrogram shows the clustering of the samples collected from pigs from newborn through 19 weeks, as well as sow tonsillar samples. Samples are color coded by week of sampling. Small legends indicate some of the challenges that took place in specific times.

We wondered if the development of the tonsillar microbiome followed a temporally dependent successional pathway and to what degree, if any, dietary antibiotics, and management pratices influenced the microbiome. An unrooted dendrogram based on a Bray-Curtis analysis (Figure 2) shows the clustering of the pig tonsillar microbiome samples from newborn through the nineteenth week. Samples from newborn piglets were mainly distributed in two groups, one corresponding to pigs from a first parity sow (4/16; 25%) and the other from pigs of multiparous sows (10/16; 62.5%), the remaining two samples were clustered with microbiome samples of older pigs. At 1 week of age, the microbiome samples were clustered by litter in four different groups.

**Figure 3:**
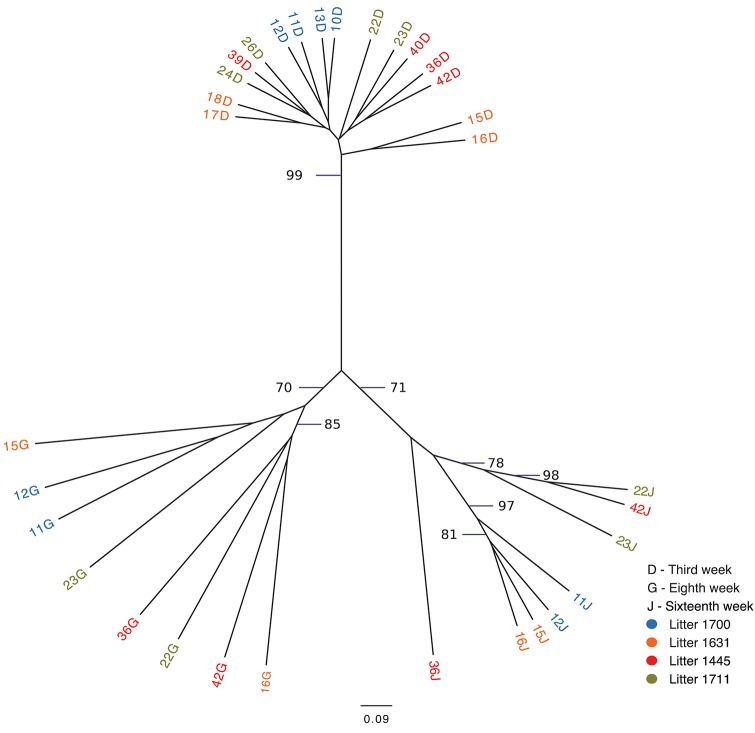
Unrooted Bray-Curtis dendrogram for three pre-transition times. The dendrogram shows the clustering of tonsil microbiome samples from pigs at three times immediately before challenging events: week 3, week 8, and week 16. Samples are color coded by week of sampling. Bootstrap values higher than 70 are shown.

The following weeks showed that as pigs aged, their tonsil microbiomes tended to become more similar. During the second week, the samples clustered together in three related groups. In the third week, all sixteen samples clustered together in one group, which also included ten of the week 2 samples. The fourth week, which marked a transitional time after a challenge, i.e., weaning plus movement to new housing plus addition of Carbadox® to the new solid feed, showed a split of the previously tightly clustered samples into four separate groups, which were not clustered by litter. The sixth week, again marked by a transition after a challenge, i.e., removal of Carbadox® and addition of Tylan® to the feed, again showed samples clustered in four separate groups, which were neither clustered by litter nor the same, with one exception, as the groups for the fourth week samples. However, for the 8 week, samples clustered in only two groups. Once again, in the tenth week, which marked the transition after a challenging condition, i.e., removal of Tylan® from feed and reassignment to new finishing rooms with litter groups broken up, showed a major disruption in the clustering pattern with samples falling into six different groups. The sampling times corresponding to weeks 12 and 16, a time of stability for the piglets, once again showed coalescing of the microbiota phylogenetic compostion; the twelfth week samples clustered into three groups, while the sixteenth week samples all clustered into a single group. Finally, for the last sampling period corresponding to the nineteenth week, also a transitional time after a challenge, i.e., movement to new finishing rooms with another re-assortment of the piglets, the samples once again showed a split into three different groups. The clustering pattern also showed that as the pigs aged, most samples clustered with tonsillar samples from sows, despite no longer having contact with the sows. Based on the above analysis we identified three sampling times (third, eighth and sixteenth weeks), which were immediately before a challenging condition, where the microbiome tended to be more similar between pigs. Statistical support for this clustering pattern is shown in an unrooted dendrogram based on Bray-Curtis analysis (Figure 3), where samples derived from the third, eighth and sixteenth weeks formed three distinct groups which were supported by bootstrap values higher than 70.

We also analyzed the clustering shown in Figure 2 to determine whether there were effects of litter or of pen on the clustering. Samples from newborn and 1 week old pigs clustered by litter, but older animals did not. We saw no correlation of the clustering with groups of piglets in the same pens except as related to the litter effect seen in newborn and 1 week old animals.

### Tonsil microbiome membership throughout the life of the pigs

**Figure 4:**
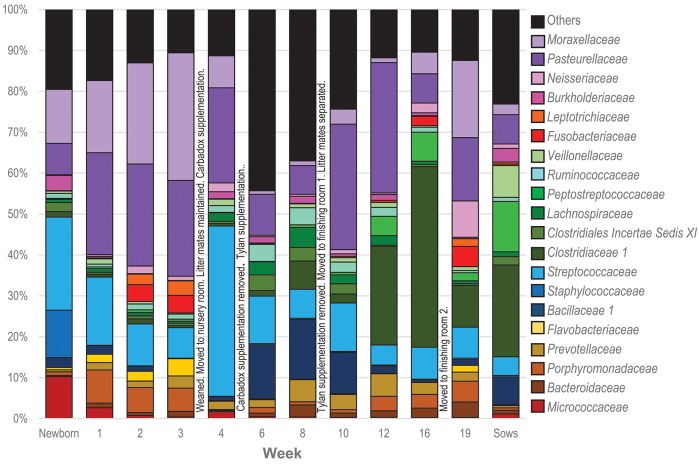
Twenty most abundant families identified in the tonsillar microbiome of pigs from newborn to market age and in the sows. Colored bars illustrate the percentage of the total reads classified into specific families at each sampling time and are the average of all animals at each time point. Text boxes in the plot indicate times when a challenging event occurred. “Others” represents members of bacterial families different from the 20 most abundant families identified.

To visualize how the membership of the tonsillar microbiome changes through the life of the pigs, we plotted the proportion of the 20 most commonly identified bacterial families in piglets at each sampling time, as well as in sows (Figure 4). Members of the phyla *Actinobacteria* (Family *Micrococcaceae*), *Bacteroidetes* (Families *Bacteroidaceae, Porphyromonadaceae, Prevotellaceae, Flavobacteriaceae*), *Firmicutes* (Families *Bacillaceae 1, Staphylococcaceae, Streptococcaceae, Clostridiaceae 1, Clostridiales Incertae Sedis XI, Lachnospiraceae, Peptostreptococcaceae, Ruminococcaceae, Erysipelotrichaceae, Veillonellaceae*), *Fusobacteria* (Family *Fusobacteriaceae*), and *Proteobacteria (*Families *Burkholderiaceae, Neisseriaceae, Pasteurellaceae*, and *Moraxellaceae*) were identified as the most abundant bacterial phyla and families in pig tonsils. The distribution and proportions of these bacterial families fluctuated through the sampling period (Table 3), with the largest shifts related with challenging conditions experienced by the pigs. Three families that consistently represented a major portion of the tonsil microbiome across all time points were the *Streptococcaceae, Pasteurellaceae*, and *Moraxellaceae*.

**Table 3:** Top 20 most abundant families at each sampling time (% of total).

	**Week**											
**Family**	**Newborn**	**1**	**2**	**3**	**4**	**6**	**8**	**10**	**12**	**16**	**19**	**Sow**
*Corynebacteriaceae*	2.02	0.14	0.07	0.07	0.22	0.68	0.68	0.28	0.10	0.25	0.30	1.50
*Micrococcaceae*	10.25	2.63	0.74	0.42	1.68	0.43	0.38	0.29	0.30	0.23	0.26	1.11
*Bacteroidaceae*	0.30	1.14	0.74	1.33	0.29	0.93	2.94	1.07	1.60	2.33	3.78	0.93
*Porphyromonadaceae*	0.85	8.11	6.11	5.69	0.21	1.34	0.78	0.81	3.54	3.34	5.11	0.59
*Prevotellaceae*	0.51	1.79	1.58	2.97	2.05	1.90	5.40	3.75	5.52	2.92	2.21	0.55
*Rikenellaceae*	0.24	0.07	0.02	0.00	0.04	1.16	0.54	0.70	0.04	0.02	0.11	0.00
*Flavobacteriaceae*	0.59	2.06	2.38	4.22	0.18	0.25	0.11	0.17	0.10	0.08	1.67	0.18
*Chitinophagaceae*	0.17	0.01	0.00	0.01	0.04	0.15	0.10	0.14	0.56	0.28	0.04	0.11
*Bacillaceae 1*	2.40	2.10	1.29	0.13	0.94	13.13	14.60	10.07	1.83	0.65	1.37	6.83
*Bacillales Incertae Sedis XI*	0.19	1.32	1.93	1.22	0.51	0.06	0.14	0.09	0.04	0.44	0.82	0.76
*Planococcaceae*	0.15	0.16	0.10	0.03	0.10	0.82	0.88	0.65	0.08	0.24	0.11	1.25
*Staphylococcaceae*	11.60	0.07	0.06	0.03	0.08	0.34	0.30	0.25	0.19	0.08	0.36	0.35
*Aerococcaceae*	0.36	0.57	0.18	0.07	0.09	0.00	0.00	0.04	0.17	0.33	1.23	0.15
*Lactobacillaceae*	0.16	0.60	0.31	0.03	2.25	0.55	0.21	0.19	0.23	0.22	0.06	0.08
*Streptococcaceae*	22.77	16.71	10.23	7.45	41.64	11.63	7.09	11.86	4.90	7.83	7.58	4.56
*Clostridiaceae 1*	1.30	0.67	1.21	0.49	0.58	1.35	6.90	2.21	24.10	44.14	10.19	22.48
*Clostridiales Incertae Sedis XI*	2.25	0.49	0.73	0.59	0.59	3.82	3.29	2.48	0.32	0.56	0.43	2.03
*Lachnospiraceae*	0.78	0.87	0.85	0.49	2.06	3.21	4.92	2.18	2.30	0.77	0.72	1.17
*Peptostreptococcaceae*	0.24	0.45	0.67	0.50	0.11	0.08	0.65	0.63	4.75	7.09	1.97	12.31
*Ruminococcaceae*	1.21	0.67	1.41	1.24	1.64	4.18	4.19	2.52	2.17	1.21	0.53	1.00
*Erysipelotrichaceae*	0.69	0.47	0.41	0.32	0.54	0.06	0.29	0.25	0.53	1.33	0.39	7.77
*Veillonellaceae*	0.59	1.28	0.61	0.28	1.57	0.27	0.93	1.05	1.20	0.39	0.95	0.39
*Fusobacteriaceae*	0.09	0.23	4.11	4.29	0.12	0.01	0.13	0.34	0.54	2.28	4.96	0.49
*Leptotrichiaceae*	0.00	0.00	2.58	3.55	0.00	0.06	0.01	0.00	0.01	0.16	1.88	0.49
*Caulobacteraceae*	2.20	0.05	0.02	0.07	0.26	0.06	0.07	0.14	0.04	0.00	0.00	0.48
*Sphingomonadaceae*	0.31	0.10	0.01	0.03	0.31	0.52	0.51	0.33	0.19	0.03	0.20	0.98
*Burkholderiaceae*	3.73	0.38	0.13	0.08	1.68	1.54	1.73	0.62	1.39	0.69	0.35	3.32
*Comamonadaceae*	0.75	0.17	0.11	0.02	0.27	0.97	1.11	0.73	0.40	0.18	0.04	0.98
*Neisseriaceae*	0.17	0.43	1.87	1.07	2.17	0.36	0.48	1.01	0.40	2.39	8.90	1.08
*Succinivibrionaceae*	0.04	0.01	0.02	0.00	0.05	0.05	1.45	0.03	0.06	0.02	0.00	0.14
*Enterobacteriaceae*	0.93	0.49	0.25	0.04	0.49	2.32	1.10	0.86	0.37	0.43	0.27	0.78
*Pasteurellaceae*	7.68	24.90	24.93	23.40	23.25	10.00	7.02	30.70	31.87	7.16	15.47	7.17
*Moraxellaceae*	13.19	17.67	24.72	31.22	7.85	0.87	1.15	3.68	1.16	5.28	18.87	2.61
*Pseudomonadaceae*	0.12	0.06	0.01	0.01	0.09	0.33	0.71	0.45	0.91	0.03	0.00	0.38
*Xanthomonadaceae*	1.15	0.16	0.05	0.01	0.23	1.47	0.95	0.67	0.34	0.11	0.04	0.36
*Spirochaetaceae*	0.23	0.22	0.47	0.11	0.08	0.56	1.39	0.57	0.33	0.35	1.50	0.81
Others	9.80	12.76	9.07	8.51	5.71	34.54	26.88	18.22	7.43	6.15	7.33	13.84

*Red, families that represent the top twenty most abundant families for a specific period or sample*.

*Blue, families that were not part of the top 20 families for that sampling period*.

*Black, other members of the microbiome not listed in the table*.

The microbiome of newborns was characterized by the abundant presence of the families *Streptococcaceae, Moraxellaceae, Staphylococcaceae*, and *Micrococcaceae*, each representing 10 to 23% of the total; members of families *Pasteurellaceae, Burkholderiaceae*, and *Bacillaceae* as well as members of the order *Clostridiales* were identified in smaller proportions. In the first week, *Pasteurellaceae* and *Porphyromonadaceae* increased dramatically, to 25 and 8.1%, respectively. *Moraxellaceae* also increased slightly, while there was a slight decrease in *Streptococacceae*. A more dramatic decrease was evident for *Staphylococcaceae*, which almost disappeared, and *Micrococcaceae*. Over the next 2 weeks, members of the *Streptococcaceae* continued to decrease, and *Micrococcaceae* virtually disappeared. In contrast, members of *Moraxellaceae* continued to increase. Members of *Pasteurellaceae* remained constant. *Fusobacteriaceae* appeared in week 2 and remained present in week 3. Multiple members of the order *Clostridiales* (*Clostridiaceae 1, Clostridiales Incertae Sedis XI, Lachnospiraceae, Peptostreptococcaceae*, and *Ruminococcaceae*) were present in proportions lower than 1%, each, throughout the first 3 weeks of life in these piglets.

The transition between the third and fourth weeks, when the piglets were weaned, moved to new housing, and shifted to solid food containing Carbadox®, was marked by drastic shifts in the tonsil microbiome. *Moraxellaceae* decreased dramatically from 31.2% in week 3 to 7.9% in week 4, *Streptococcaceae* bloomed from 7.4 to 41.6%, while *Pasteurellaceae* and *Clostridiales* remained steady. Members of *Fusobacteriaceae* and *Porphyromonadaceae* almost disappeared.

Week 6, after another major transition when Carbadox® was removed from feed and Tylan® added, was again marked by drastic shifts in the tonsil microbiome. Overall sobs, as described above, increased from 111 to 368 (Table 2), indicating a massive increase in diversity. Members of the *Streptococcaceae* and *Pasteurellaceae* both decreased dramatically, from 41.6 to 11.6% and 23.2 to 10%, respectively, and *Moraxellaceae* decreased and almost disappeared. However, members of *Bacillaceae 1* and some members of the order *Clostridiales* (*Clostridiales Incertae Sedis XI, Lachnospiraceae*, and *Ruminococcaceae*) began to flourish and increased substantially, particularly *Bacillaceae* 1 which increased from 0.9 to 13.1%. Interestingly, almost 44% of the members of the tonsillar microbiome did not fit into these twenty most abundant bacterial families for this time point, again indicative of an overall increase in diversity.

In the eighth week, the decreasing trend for *Streptococcaceae* and *Pasteurellaceae* continued and each family dropped to a relative abundance of 7%. *Moraxellaceae* remained in very low abundance. However, anaerobic organisms including *Clostridiales*, particularly *Clostridiaceae 1*, and *Bacteroidales* increased. The proportion of identified bacterial families that were not included in the twenty most abundant was still close to 40%.

The tenth week, which corresponded to another significant transition period for the pigs, i.e., removal of Tylan® from feed as well as movement to finishing rooms and reassortment of litter members, was again marked by a major shift in the microbiome. The three predominant families, *Pasteurellaceae, Streptococcaceae*, and *Moraxellaceae*, all increased, particularly the *Pasteurellaceae* that increased from 7% to 30.7%. In contrast, members of the *Clostridiales* (*Clostridiaceae 1, Clostridiales Incertae Sedis XI, Lachnospiraceae*, and *Ruminococcaceae*) and *Prevotellaceae* decreased, as did the proportion of the microbiome classified as “Others.”

Over the next 6 weeks, represented by sampling times at 12 and 16 weeks, the tonsil phylogenetic structure of the microbiome in all of the pigs coalesced to a common core (Figure 2). Overall, there was a massive increase in the *Clostridiales*, particularly *Clostridiaceae 1*, and *Peptostreptococcaceae*, from 2.2 and 0.6% in week 10 to 44.1% and 7%, respectively in week 16. Over the same period, *Pasteurellaceae* decreased from 30.7 to 7.1%, and *Bacillaceae 1* decreased from 10.1% to 0.6%. *Streptococcaceae, Moraxellaceae*, and *Bacteroidales* remained relatively stable. The proportion of identified bacterial families that were not included into the twenty most abundant families decreased to ~10%. By week 16, *Fusobacteriaceae* and *Neisseriaceae* reappeared in low proportions.

Finally, the nineteenth week, which coincided with a transitional period in which penmates were reassorted into new rooms, was marked by another significant disruption of the microbiome. Overall, an increase in *Pasteurellaceae, Moraxellaceae, Neisseriaceae*, and *Fusobacteriaceae* was paired with a dramatic decrease in *Clostridiales*, particularly *Clostridiaceae 1* and *Peptostreptococacceae*.

**Figure 5:**
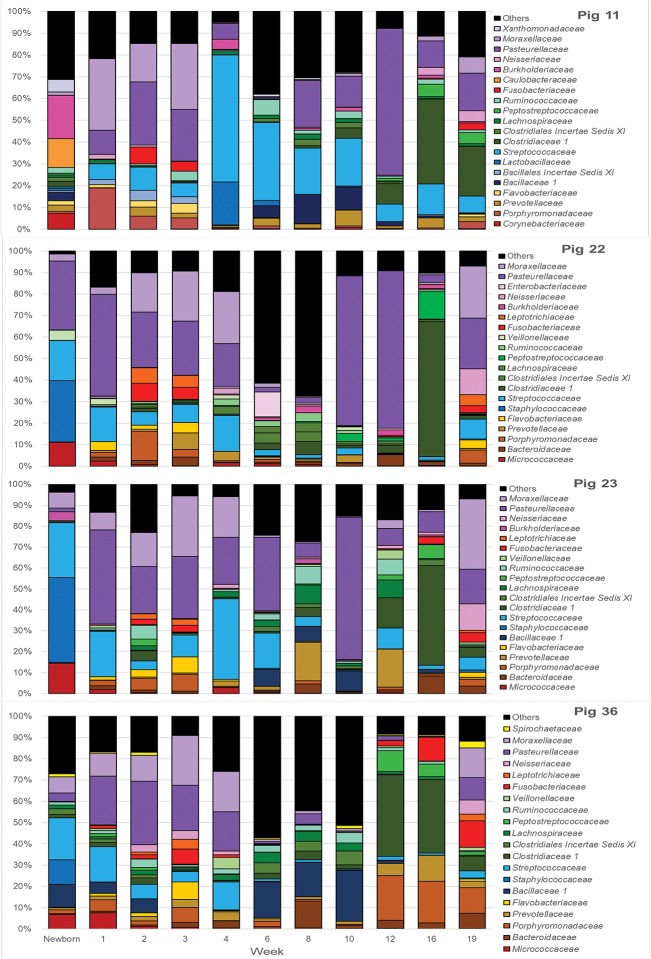
Twenty most abundant families in the tonsilar microbiome per sampling time for 4 selected pigs. Colored bars illustrate the percentage of the total reads classified into specific families at each sampling time from newborn through market age for pig 11, pig 22, pig 23, and pig 36.

It should be noted that, after the first 3 weeks and weaning, these shifts in the microbiome were not synchronous in all piglets. Figure 2 shows several clusters that contain samples from sequential weeks, e.g., weeks 6, 8 and 10; weeks 8, 10, and 12; and weeks 10, 12, and 16, indicating that common microbiomes, represented by the clusters, were reached at different times in different pigs. This is further illustrated in Figure 5, which shows the most abundant microbial families over time in 4 different pigs. As examples, a microbiome with a preponderance of *Pasteurellaceae* is seen in week 10 in pig 23, week 12 in pig 11, both weeks 10 and 12 in pig 22, and not at all in pig 36. A microbiome with a preponderance of *Clostridiaceae*, mainly *Clostridiaceae 1* and *Peptostreptococcaceae*, is seen in weeks 12 and 16 in pig 36, week 16 in pigs 22 and 23, and weeks 16 and 19 in pig 11.

The tonsillar microbiome of sows was dominated by members of *Clostridiaceae 1* (~23%) and *Peptostreptococcaceae* (~12%). Other families present in proportions between 1 and 8% included *Erysipelotrichaceae, Pasteurellaceae, Bacillaceae 1, Streptococcaceae, Burkholderiaceae, Moraxellaceae, Neisseriaceae*. *Micrococcaceae*, and other members of the *Clostridiales* (*Clostridiales Incertae Sedis XI, Lachnospiraceae*, and *Ruminococcaceae*).

### Distribution of specific OTUs throughout the life of the pigs

**Figure 6:**
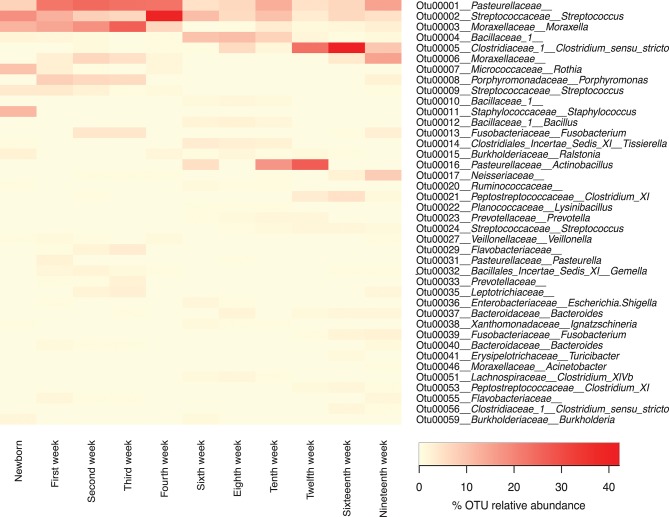
Forty most abundant Operational Taxonomic Units (OTUs) for pigs through different sampling times. Heat-map showing the relative abundance of the top 40 OTUs identified per sampling time through the life of these pigs. Note that any OTUs identified as “unclassified” at the family level were not included in this list.

While presentation of the microbiome data at the taxonomic level of family gives the best overview of the data over time, we also examined the presence and abundance of specific OTUs over time (Figure 6). At the family level, *Pasteurellaceae, Streptococcaceae*, and *Moraxellaceae* predominate throughout the life of the pigs. However, within the top 40 OTUs there were three OTUs of *Pasteurellaceae* seen, including OTU0001, which was present in high concentration during weeks 1–4 but never lost, OTU0016 which appeared in weeks 6 and 10–12; and OTU0031, which was mainly seen in 1 week old piglets. Similarly, there were three OTUs of *Streptococcaceae*, including OTU002 which was seen throughout the lives of the pigs but was particularly dominant in week 4, OTU009 which was seen in newborns and weeks 1–4, and OTU0024, which was seen mainly in older piglets. Finally, there were three OTUs of *Moraxellaceae*, including OTU003, which was a major component of the microbiome in newborns through week 4 and then decreased to return in the week 10 and 19 samples; OTU0006, which was present in lower amounts than OTU0003 in weeks 1–4 but in much higher amounts in weeks 16 and 19; and OTU0046, which was a minor component of the microbiome.

### Aerobic, anaerobic, and facultatively anaerobic organisms in the tonsils

**Figure 7:**
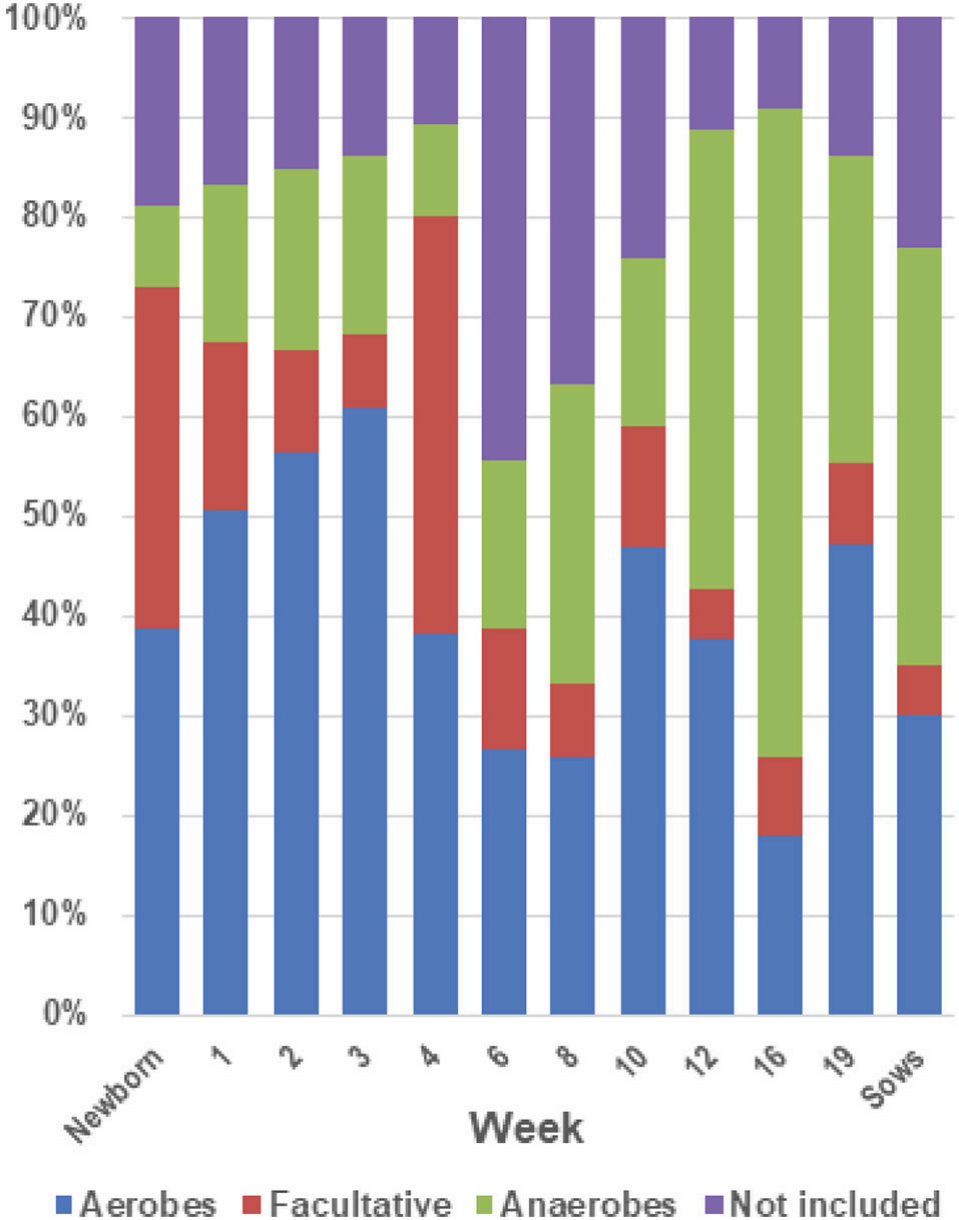
Proportions of aerobes, anaerobes and facultative bacteria in the tonsil microbiome. The twenty most abundant families identified in the tonsillar microbiome of the sampled pigs from newborn through market age, as well as sows, were classified as aerobes, anaerobes or facultative organisms based on Bergey's Manual of Systematic Bacteriology (28). The chart illustrates the proportion of the microbiome classified as aerobes, anaerobes, and facultative anaerobes at each sampling time.

An analysis of the distribution of the bacterial families identified in the tonsils based on their classification by use of oxygen as aerobes, anaerobes or facultative anaerobes (Figure 7) showed that in piglets aged newborn to 4 weeks the microbial population was comprised of ~70% aerobes and facultative anaerobes. The abundance of facultative anaerobes decreased from birth through week 3, but increased after weaning, most likely due to the bloom in *Streptococcaceae*. The proportion of anaerobes increased after the weaning period, with a concomitant decrease in facultative anaerobes and aerobes, and reached ~65% of the total microbiome in week 16.

## Discussion

We have previously characterized the tonsil bacterial microbiome in healthy 18–20 week old grower-finisher pigs (14) and have recently described the development of the tonsillar microbiome in pigs from birth to weaning (16). In this study, we sought to extend this research to characterize how that tonsil microbial community develops and matures during the life of pigs from newborn to market age. In particular, we wished to determine when specific members of the tonsil microbial community appeared and disappeared, whether there was a temporal succession in the development of the community, and whether stressful events such as alteration of feed or housing, addition or removal of in feed antibiotics, or mixing of pigs into new social groups affected the structure and composition of the tonsillar microbiome. Although clearly there are other microbes such as fungi and viruses present in porcine tonsils, we focused on the bacterial communities in this study.

There are strong parallels between our current data and that from the prior study on 18–20 week old pigs (14). In both studies, members of the tonsil microbiome were found to predominantly belong to 5 phyla: *Proteobacteria, Firmicutes, Bacteroidetes, Fusobacteria*, and *Actinobacteria*, with *Proteobacteria* and *Firmicutes* together representing 85-90% of the tonsil microbiome. In both studies, *Pasteurellaceae, Moraxellaceae, Neisseriaceae, Streptococcaceae, Peptostreptococcaceae, Veillonallaceae*, and *Fusobacteriaceae* were identified as among the most abundant bacterial families found. In the current study, many families in the orders *Clostridiales* and *Bacteroidetes* were also found to be among the most abundant taxa seen. Improvements in both the sequencing technology and the databases that facilitate identification of bacteria via 16s rRNA gene sequencing likely account for these differences. In the earlier study, it was not possible to identify most *Clostridiales* below the order level, which is now possible. Further, in that study it was recognized that *Bacteroidetes* were underrepresented in the final data, possibly due to amplification bias with the primers used in that study (14).

We collected samples from eleven different sampling periods from newborn to 19 weeks of age (Figure 1) as well as the tonsillar microbiome of the sows. In our analysis of the taxa (at the family level and the OTU level) in these samples, we observed that the development of tonsillar communities in pigs followed a successional process. Some members of the community were acquired during the birth process, from the sow vaginal tract, or within the first few hours of life from the sow teat skin or milk (16), while others were acquired later. Some members of the community, particularly *Streptococcaceae, Pasteurellaceae*, and *Moraxellaceae*, were present throughout the life of the pigs, while others such as *Staphylococcaceae, Micrococcaceae*, and *Fusobacteriaceae* seemed to be transient (Figure 4). Further, specific OTUs of some of these major taxa also appeared to be either permanent or transient (Figure 6). The relative proportions of the major members of the microbiome did change through time, however, as pigs aged, their microbiome seemed to become more similar to the microbiome of older pigs (Figure 4 and Table 3). This process was not always synchronous (Figure 5), but the overall progression was very similar in most pigs.

As we examined the changes in the microbiome over time, it became clear that at certain time points, e.g., at 3, 8, and 16 weeks, the phylogenetic structure of the microbiomes of all of the animals became very similar (Figures 2, 3 and Table 2). When we analyzed this in comparison to management of the pigs (Figure 1), we concluded that stretches of time with constant conditions, such as newborn through week 3, led to the convergence of microbiome structure across samples. This convergence occurred regardless of litter source for the pigs or room in which they were housed. In contrast, times where there were changes in management conditions, such as addition or removal of in feed growth promoting antibiotics or movement of pigs to new housing, and especially weaning, led to perturbations in the microbiome (e.g., weeks 4, 6,10, and 19). The taxonomic data was supported by an analysis of the alpha diversity (Table 2). Whether these perturbations occurred in response to specific stresses, such as presence of antibiotics, or were adaptations of the microbiome to new conditions, such as new feed, or resulted from exposure to new microorganisms when groups of piglets were reasserted to new rooms, or a simultaneous combination of such stresses, remains unclear. However, absence of challenges or disruptions led to stabilization of the microbiome, with most pigs developing similar microbiomes over times with constant conditions.

Our recent study that followed the development of the tonsillar microbiome of piglets from newborn to weaning, focusing on the source of members of the microbiome and the litter effect as well as the overall development and the effect of weaning (16), and the current study that extends that work, are the only studies available that describe the development of the tonsillar microbiome of pigs or other mammals. Most of the available data following the development of microbial communities in mammals has been focused on the gastrointestinal tract. Pajarillo et al (29) assessed the fecal bacterial diversity of healthy piglets during the weaning transition, and suggested that this period was related to a trend of increasing bacterial diversity, which may be related with the changes in diet. However, they did not discard a possible additional influence of stress or disruption associated with the weaning period. Another study describing the bacterial diversity of pig feces over time followed the development of the fecal microbiome of pigs 10–22 weeks old and identified that calculated diversity indices suggested similar diversity profiles for all the samples (30). Although these prior studies examined the fecal microbiome, they support our results of increased bacterial diversity when the piglets were weaned, which decreased after 10–12 weeks, as well as following challenges or disruptions, such as addition or removal of in feed antibiotics.

There is extensive research data showing that the balance of microbial communities is altered by the use of antibiotic treatments (22, 31). Rettedal et al. (32) studied the effect of the growth promoter chlortetracycline on the ileal microbiota of pigs and found an association with a significant shift in the gut microbiota. However, Poole et al. (33) did not find changes in diversity in feces associated with a similar dose of chlortetracycline. In-feed supplementation of pigs with a mixture of antibiotics known as ASP250, containing chlortetracycline, sulfamethazine, and penicillin, was correlated with a shift in the bacterial phylotypes present in the intestine, where microbial community membership changed over time, mainly showing a decrease in *Bacteroidetes* abundance and an increase in *Proteobacteria* (34). Carbadox® supplementation in-feed was associated with significant changes in community structure and bacterial membership in the intestinal microbiota of pigs (35). An immediate effect was noticed, although the microbiome structure recovered later despite the continued use of the medication. The authors reported a relative increase in *Prevotella* associated with Carbadox® administration, while Carbadox® withdrawal was associated with an increase in the *E. coli* population (35). The use of the growth promoter Tylosin (also known as Tylan®) was associated with a pronounced shift in the intestinal microbiome distribution and quantity, altering the abundance of specific genera such as *Lactobacillus* among others. These changes occurred at specific times in the growing pig as the pigs aged (36). These prior studies have tried to identify the effects of medicated food on the gut/feces microbiome, but there are no studies that characterize the effect of ingested medications on the tonsillar microbiome. However, it can be concluded that regardless of the medication, the administration of antibiotics or growth promoters in food exerts an effect on the bacterial communities. In this study, we observed large shifts in the tonsil microbiome related to specific periods where medicated food was added, changed or removed. However, because in feed medication was not an isolated factor but supplementary to other changes at the same time, we cannot make a definitive conclusion about the specific effect of the administration of this medication. We do consider the microbiome shifts seen to be relevant and the potential subject of further research.

We identified the first major shift associated with supplementation of Carbadox® coinciding with a huge bloom in members of *Streptococcaceae* and a decrease in *Moraxellaceae, Fusobacteriaceae*, and *Porphyromonadaceae*, reported previously by our group (16). Another shift was associated with the removal of Carbadox® and supplementation with Tylan®, with a major decrease in members of *Streptococcaceae, Moraxellaceae*, and *Pasteurellaceae* with a concurrent increase in members of *Clostridiales* and *Bacillaceae 1*. The removal of Tylan® from the diet was associated with a slight increase in members of *Streptococcaceae* and *Moraxellaceae*, parallel to a higher increase in members of *Pasteurellaceae*. It is important to highlight that the presence of Tylan® in the diet is associated with an increase in the bacterial diversity (Table 2 and Figure 4). We emphasize that our goal was not directed toward the identification of specific effects of antibiotics or growth promoters in the development of the tonsil microbiome, but instead toward characterization of the development of tonsillar microbiome of pigs from a healthy farm under normal management. Our results open an avenue for future research on the specific effect of these medications on the tonsillar microbiome and how they can potentially influence the acquisition of pathogenic flora.

Another big change experienced by the pigs was dietary, particularly at weaning. In human infants, the introduction to a new diet associated with cessation of breast feeding has been shown to be associated with profound changes in the composition of the intestinal microbiome (37). It has been suggested that the diet to which an individual has been exposed rapidly alters the structure of the intestinal microbial communities (38). Similarly, in pigs it has been shown that the diet can have an effect in the intestinal microbiome (39), and in particular that the diet supplemented after weaning in piglets can alter the fecal microbiota considerably. A diet supplemented with fermentable carbohydrates was related with greater bacterial diversity when compared to control diets (39). Diet changes during weaning transition can exert an effect on the composition of the intestinal microbiota (40, 41) where bacterial community structure can change as the diet changes (42).

It is not clear whether the bloom in *Streptococcaceae* seen at 4 weeks was related to weaning and removal from the sows, supplementation with Carbadox^®;^, or both. The most common OTU of the *Streptococcaceae* in this bloom was identified as *Streptococcus suis*, an organism that is both normal microbiota of swine tonsils and a cause of severe infections including meningitis and polyarthritis in recently weaned piglets (43, 44). *S. suis* is also an emerging pathogen of humans (43, 45). Attempts to eradicate *S. suis* from pig herds by segregated early weaning were not successful, likely due to transmission of this organism during birth from the sow vagina tract to the oropharynx of piglets (16, 46). Many swine farms employ in feed antibiotics to reduce problems with *S. suis*. Our data suggest that Carbadox® is not effective and indeed may exacerbate the problem.

Finally, the environmental changes experienced by the pigs could play a role in the development of the tonsillar microbiome. These can include both changes in the physical environment and exposure to new penmates after reassortment of pigs into new housing. The immediate environment in which pigs grow has been suggested to have a profound influence on the initial acquisition and development of fecal and colonic microbiota (47). A recent study following the development of gut microbiota and the effect of early changes in the environment demonstrated that microbial diversity was disturbed by changes in environmental hygiene, and that the effect of the generated changes remained for a long time in the affected animals (8). In our study, we saw increased fecal anaerobes, such as *Clostridiales*, in the tonsils (Figures 4, 7) after weaning and especially after Tylan® was removed from feed. Pigs are coprophagic, and it is likely that these anaerobes were acquired from ingestion of feces from the pen floors. In the older pigs, crypt abscesses in the deeper areas of elongating crypts might provide a niche for colonization by the acquired anaerobes, or conversely these anaerobes may cause the formation of the crypt abscesses. We have previously observed that pigs housed in a very clean high biosecurity environment had almost no *Clostridiales* in the tonsils (unpublished data). Conversely, the absence of deep crypts in very young pigs as seen by scanning electron microscopy suggests an absence of appropriately anaerobic sites within young tonsils for colonization by anaerobes (48).

In the sixteenth week, members of *Clostridiales, especially Clostridiaceae 1*, and *Peptostreptococcaceae*, comprised ~51% of the identified members of the microbiome for this period. Our results compare with those of Bokulich et al. (31), who studied the development of fecal microbiota in children during early life and associated the administration of antibiotics in children during first months of life with a deficit in members of the *Clostridiales*. Further, the authors associated a gradual increase in members of this order with the introduction to solid food. Our findings become especially relevant when compared with recent findings reported by Kim et al. (7), which found that the presence of members of *Clostridiales* in the enteric microbiota of mice is critical to prevent the growth of enteric pathogens in the intestine. We do not know how this finding can be translated to the tonsillar microbiome of pigs, but it is interesting to see that one of the most vulnerable periods for pigs to acquire diseases (weaning through eighth week) was marked by a low abundance of members of the *Clostridiales*.

In this study, we found that some bacterial families dominated the tonsillar microbiome throughout the life of pigs; however, their relative abundance often changed significantly after the challenging events. Similarly, other bacterial families appeared and/or disappeared at specific ages. A longitudinal study of bacterial diversity in feces of commercial pigs found that some phyla dominated the microbiome regardless of the age of the animals, supporting our findings in the tonsils. Further, it was observed that a small group of organisms were the most prevalent microbes as pigs aged, and their microbiome converged with the time when they were maintained under similar conditions (30). Although this study was focused on the fecal microbiome, it supports our results in the development of tonsillar microbiome, where we identified some bacterial families that dominated and were present throughout the study period, as well as other bacterial families that were transient and appeared at different times, and further saw a convergence of the tonsil microbiome in all the pigs when they were maintained under constant conditions.

Jensen et al. (11) characterized the microbiome of tonsillar crypts of human patients either with chronic tonsillitis or tonsils from healthy patients which were removed because of hyperplasia. The authors could identify a core microbiome population at the species level in the crypts of humans independent of their health status and age, which involved the genus *Streptococcus, Prevotella, Fusobacterium, Porphyromonas, Neisseria, Parvimonas, Haemophilus, Actinomyces, Rothia, Granulicatella*, and *Gemella*. The above identified genera are members of the families *Streptococcaceae, Prevotellaceae, Fusobacteriaceae, Porphyromonadaceae, Neisseriaceae, Clostridiales Incertae Sedis XI, Pasteurellaceae, Actinomycetaceae, Micrococcaceae, Carnobacteriaceae*, and *Bacillales Incertae Sedis XI*, respectively. Similarly, other studies identifying the human microbiome have recognized members of families *Streptococcaceae, Prevotellaceae*, and *Fusobacteriaceae* as abundant in the tonsillar microbiome of healthy humans (10, 17, 49). Although we did not characterize specifically the microbiome of tonsillar crypts and we were not able to characterize the members of the community further than family or genus level for some taxa, our results also show that members of the above mentioned families, except *Actinomycetaceae* and *Carnobacteriaceae*, comprised some of the most abundant families identified in pig tonsils. It was found that members of the genus *Staphylococcus* were present only in low proportions in human tonsils (11). Similarly, we identified that members of the family *Staphylococcaceae* were abundant only in the newborns and decreased noticeably and almost disappeared in the following weeks.

A Bray-Curtis analysis of the development of the pig tonsil microbiome from birth to market age (Figure 2) showed us that as the pigs were getting older, the acquired microbial population tended to be more similar to the microbiome present in adult pigs, i.e., the tonsillar microbiome of sows (Figure 4). We identified that between the sixth to tenth week, some samples clustered with a sample from the tonsillar microbiome of sows. However, a higher percentage of samples from older pigs, especially between twelfth to nineteenth weeks, were clustered together with samples from tonsillar microbiome of sows. These findings demonstrate both that there is a succession in the development of tonsillar microbiome in pigs and that the final status of the microbiome in grower/finisher pigs develops to resemble that of adult animals. Similar findings were reported by other authors studying the development of the human intestinal microbiota (20, 31), which found that as infants aged, their gut microbiome began to look like the adult microbiome, although it did not reach a mature stage found in adults. Our results show that although the microbiome of older pigs was more similar to the microbiome of the sows, there are still observable differences in the abundance of certain families, as the case of members of families *Peptostreptococacceae, Erysipelotrichaceae* and *Burkholderiales* which were more prominent in sow microbiome. Many other studies have also shown that there is a succession/sequentiality in the development of microbial communities in mammalian tissue (18, 21, 30, 50, 51).

In conclusion, this study provides baseline information on the development of the tonsillar microbiome of piglets from newborn to market age, as well as the tonsillar microbiome of sows. We demonstrate that there was a succession in the development of the tonsillar microbiome of piglets as they age, which was not synchronous on all pigs but was highly similar. The tonsil microbiome tended to stabilize and become very similar in all animals over times where management conditions were constant. However, the challenges associated with management procedures typical in a swine farm generated prominent changes in the microbiome composition and the abundance of diverse bacterial families. Nonetheless, over time the microbiome of these young pigs tended to be more similar to the microbiome of older animals. We do not know if the observed patterns would be similar for all pigs from this farm, or if the same pattern would be observed independent of the breed or the specific farm. This study lays the baseline for future research to examine the effect of specific conditions, such as use of antibiotics, on the development of the tonsil microbiome and of acquisition of specific pathogens on the tonsil microbiome and conversely of the effect of the composition and structure of the tonsil microbiome on acquisition of pathogens. Manipulation of the tonsil microbiome to provide enhanced resistance to acquisition and carriage of pathogens is a potential outcome of these studies.

## Author contributions

LP, MM, and JF conceived and designed the study. LP, RL, JF, and MM collected the samples. LP and RL processed the samples. LP, TM, RL, and MM performed the data analysis and interpretation. LP, TM, and MM drafted the manuscript. All authors revised and edited the manuscript, gave final approval for the version to be published, and agreed to be accountable for all aspects of the work.

### Conflict of interest statement

The authors declare that the research was conducted in the absence of any commercial or financial relationships that could be construed as a potential conflict of interest.
